# [1,1′-Bis(di­phenyl­phosphan­yl)cobalto­cenium-κ^2^ *P*,*P*′](η^5^-cyclo­penta­dien­yl){2-[4-(4-ethynylphen­yl)phen­yl]ethynyl-κ*C*}ruthenium(II) hexa­fluorido­phosphate

**DOI:** 10.1107/S1600536813028195

**Published:** 2013-10-19

**Authors:** Ling-Zhen Zeng, Yun-Ying Wu, Guang-Xuan Tian, Zhen Li

**Affiliations:** a College of Chemistry and Chemical Engineering, Yunnan Normal University, Kunming 650500, People’s Republic of China; b College of Resources and Environment, Yuxi Normal University, Kunming 653100, People’s Republic of China

## Abstract

In the title compound, [CoRu(C_5_H_5_)(C_16_H_9_)(C_17_H_14_P)_2_]PF_6_, the Ru^II^ atom is coordinated by a cyclo­penta­dienyl ring in an η^5^-mode, one C atom from a 4,4′-diethynyl-1,1′-biphenyl ligand and two P atoms from a chelating 1,1′-bis­(di­phenyl­phosphan­yl)cobaltocenium ligand, giving a three-legged piano-stool geometry. In the crystal, weak C—H⋯F hydrogen bonds link the complex cations and hexa­fluorido­phosphate anions into a three-dimensional supra­molecular structure.

## Related literature

For the synthesis of related compounds, see: Blackmore *et al.* (1971 ▶); Oshima & Suzuki (1984 ▶); Wu *et al.* (2006 ▶). For the properties of related compounds, see: Domazetis *et al.* (1980 ▶); Gaughan *et al.* (1972 ▶); Nombel *et al.* (1999 ▶); Ziolo & Dori (1968 ▶). For related structures, see: Bruce *et al.* (2003 ▶); Hembre *et al.* (1996 ▶).
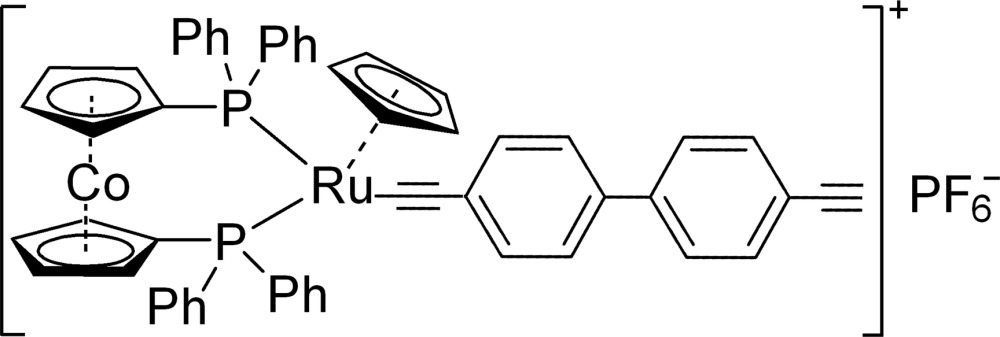


## Experimental

### 

#### Crystal data

[CoRu(C_5_H_5_)(C_16_H_9_)(C_17_H_14_P)_2_]PF_6_
*M* *_r_* = 1069.80
Monoclinic, 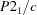

*a* = 14.481 (5) Å
*b* = 22.052 (7) Å
*c* = 14.482 (5) Å
β = 92.937 (2)°
*V* = 4619 (3) Å^3^
*Z* = 4
Mo *K*α radiation
μ = 0.85 mm^−1^
*T* = 292 K
0.30 × 0.20 × 0.20 mm

#### Data collection

Bruker APEX CCD diffractometer
Absorption correction: multi-scan (*SADABS*; Sheldrick, 1996 ▶) *T*_min_ = 0.784, *T*_max_ = 0.848
53044 measured reflections
10985 independent reflections
7027 reflections with *I* > 2σ(*I*)
*R*_int_ = 0.124

#### Refinement

*R*[*F* ^2^ > 2σ(*F* ^2^)] = 0.057
*wR*(*F* ^2^) = 0.140
*S* = 0.97
10985 reflections
595 parameters
H-atom parameters constrained
Δρ_max_ = 0.83 e Å^−3^
Δρ_min_ = −0.82 e Å^−3^

### 

Data collection: *SMART* (Bruker, 2007 ▶); cell refinement: *SAINT* (Bruker, 2007 ▶); data reduction: *SAINT*; program(s) used to solve structure: *SHELXS97* (Sheldrick, 2008 ▶); program(s) used to refine structure: *SHELXL97* (Sheldrick, 2008 ▶); molecular graphics: *XP* in *SHELXTL* (Sheldrick, 2008 ▶); software used to prepare material for publication: *SHELXTL*.

## Supplementary Material

Crystal structure: contains datablock(s) I, new_global_publ_block. DOI: 10.1107/S1600536813028195/hy2638sup1.cif

Structure factors: contains datablock(s) I. DOI: 10.1107/S1600536813028195/hy2638Isup2.hkl

Additional supplementary materials:  crystallographic information; 3D view; checkCIF report

## Figures and Tables

**Table 1:** Hydrogen-bond geometry (Å, °)

*D*—H⋯*A*	*D*—H	H⋯*A*	*D*⋯*A*	*D*—H⋯*A*
C2—H2⋯F5^i^	0.98	2.43	3.189 (6)	134
C3—H3⋯F6^ii^	0.98	2.54	3.383 (6)	144
C7—H7⋯F4^i^	0.98	2.47	3.383 (7)	155
C9—H9⋯F2^ii^	0.98	2.32	2.993 (6)	125

Symmetry codes: (i) 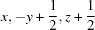; (ii) 
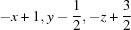
.

**Fig. 1.**
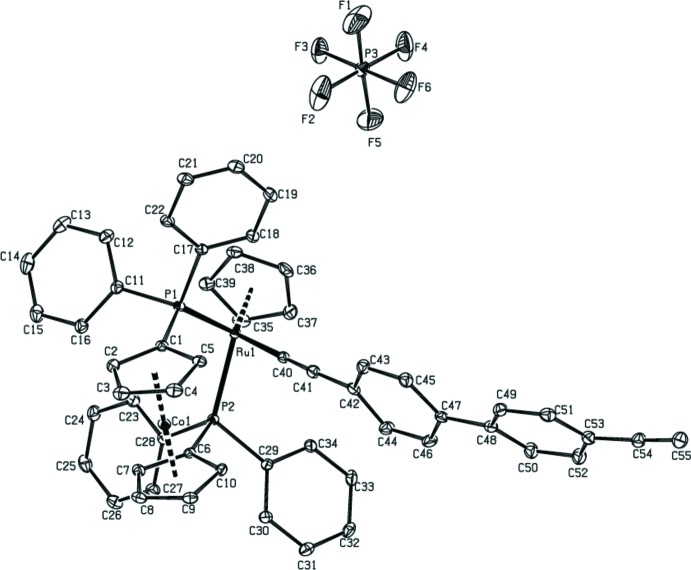
The molecular structure of the title compound, with the 30% probability displacement ellipsoids.

**Fig. 2.**
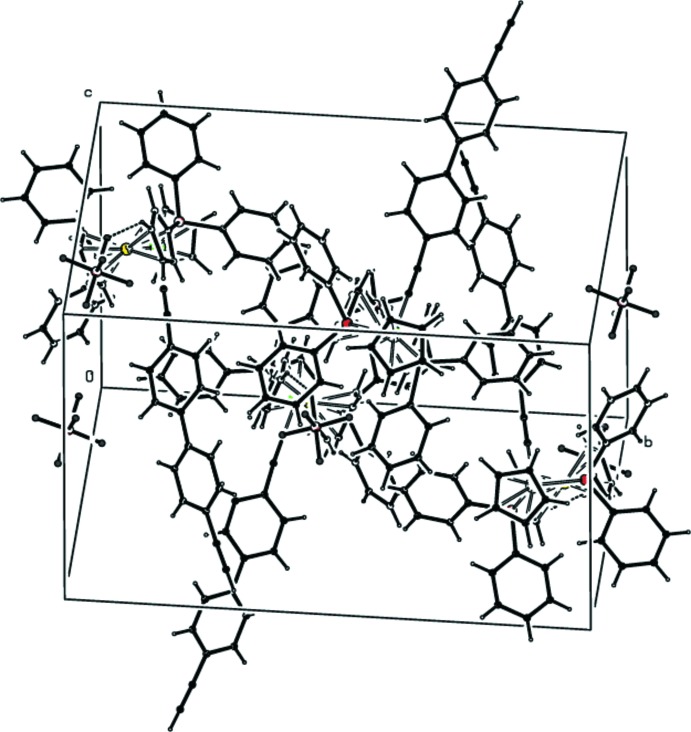
A view of the crystal packing. Hydrogen bonds are shown as dashed lines.

## supplementary crystallographic information

### 1. Comment

The fragment [Cp^*^Ru(PPh_3_)_2_Cl] (Cp^*^ = cyclopentadienyl) plays an important role in the development of organo-ruthenium chemistry and has been used as a versatile starting material for other compounds due to its stability and facile manipulation (Blackmore *et al.*, 1971; Oshima & Suzuki, 1984; Wu *et al.*, 2006). The interest in this fragment and related derivatives concerns its ability to act as a catalyst in a variety of reactions, such as decarbonylation of both aromatic and aliphatic aldehydes (Domazetis *et al.*, 1980) and hydroformylation reactions (Nombel *et al.*, 1999), as well as anti-Markovnikov hydration of terminal alkynes (Gaughan *et al.*, 1972; Ziolo & Dori, 1968). Herein we report the synthesis and structure of the title compound.

The structure of the title compound is shown in Fig. 1. The Ru—P bond lengths in the compound [2.2497 (12) and 2.2924 (12) Å] are slightly different from those in the neutral complexes, Cp^*^Ru(dppm)Cl [2.282 (2) and 2.294 (2) Å; dppm = bis(diphenyphosphanyl)methane] and Cp^*^Ru(dppe)Cl [2.2882 (5) and 2.2812 (5) Å; dppe = bis(diphenylphosphanyl)methane] (Bruce *et al.*, 2003). The Ru—C(Cp^*^) bond lengths ranging from 2.222 (4) to 2.249 (4) Å are approximately the same with those in Cp^*^Ru(dppm)Cl and Cp^*^Ru(dppe)Cl, while Ru—C(4,4'-bisethynyl-1,1'-biphenyl) bond length [2.022 (4) Å] is slightly shorter. The Ru—*Cg*1, Co—*Cg*2 and Co—*Cg*3 distances are 1.9050 (2), 1.6225 (2) and 1.6245 (2) Å (*Cg*1 is the centroid of the Cp^*^ ring, and *Cg*2 and *Cg*3 are the centroids of C1–C5 ring and C6–C10 ring), which are not significantly different from those found in Cp^*^Ru(dppf)H [dppf = 1,10-bis(diphenylphosphanyl)ferrocene] (Hembre *et al.*, 1996). The P—Ru—P angle [99.30 (4)°] in the compound is larger than those found in the dppm and dppe complexes [71.53 (6) in Cp^*^Ru(dppm)Cl and 82.15 (2)° in Cp^*^Ru(dppe)Cl], possibly due to the steric demand of the chelating dppc [dppc = 1,10-bis(diphenylphosphanyl)cobaltocenium] ligand in the title compound. The *Cg*2—P1—P2—*Cg*3 torsion angle is 5.54 (6)°. The C1–C5 and C6–C10 rings are arranged close to a synperiplanar eclipsed conformation, with a dihedral angle of 2.0 (2)°. The dihedral angle is 19.3 (2) between the benzene rings of the 4,4'-bisethynyl-1,1'-biphenyl ligand, which are not coplanar. In the crystal, weak C—H···F hydrogen bonds link the complex cations and hexafluoridophosphate anions into a three-dimensional supramolecular structure (Table 1 and Fig. 2).

### 2. Experimental

To a solution of [Cp^*^Ru(dppc)Cl](PF_6_) (0.9 g, 1.0 mmol) in 20 ml of CH_3_OH was added 4,4'-bis[(trimethylsilyl)ethynyl]-1,1'-biphenyl (0.17 g, 0.5 mmol) in CH_2_Cl_2_ (20 ml). The mixture was refluxed for 24 h. After removal of the solvent *in vacuo*, the desired product was chromatographed on alumina by elution with CH_2_Cl_2_/CH_3_COCH_3_ (10:1) to yield 0.23 g (43%) of a green solid (Oshima & Suzuki, 1984). The single crystals were obtained by the slow diffusion of n-hexane into a dichloromethane solution of the compound.

### 3. Refinement

H atoms were positioned geometrically and refined as riding atoms, with C—H = 0.93 and 0.98 Å and *U*_iso_(H) = 1.2*U*_eq_(C).

### Crystal data

**Table:**

[CoRu(C_5_H_5_)(C_16_H_9_)(C_17_H_14_P)_2_]PF_6_	*F*(000) = 2168
*M**_r_* = 1069.80	*D*_x_ = 1.538 Mg m^−^^3^
Monoclinic, *P*2_1_/*c*	Mo *K*α radiation, λ = 0.71073 Å
Hall symbol: -P 2ybc	Cell parameters from 9162 reflections
*a* = 14.481 (5) Å	θ = 2.2–24.8°
*b* = 22.052 (7) Å	µ = 0.85 mm^−^^1^
*c* = 14.482 (5) Å	*T* = 292 K
β = 92.937 (2)°	Block, black
*V* = 4619 (3) Å^3^	0.30 × 0.20 × 0.20 mm
*Z* = 4	

### Data collection

**Table:**

Bruker APEX CCD diffractometer	10985 independent reflections
Radiation source: fine-focus sealed tube	7027 reflections with *I* > 2σ(*I*)
Graphite monochromator	*R*_int_ = 0.124
φ and ω scans	θ_max_ = 28.0°, θ_min_ = 1.4°
Absorption correction: multi-scan (*SADABS*; Sheldrick, 1996)	*h* = −19→19
*T*_min_ = 0.784, *T*_max_ = 0.848	*k* = −29→29
53044 measured reflections	*l* = −19→19

### Refinement

**Table:**

Refinement on *F*^2^	Primary atom site location: structure-invariant direct methods
Least-squares matrix: full	Secondary atom site location: difference Fourier map
*R*[*F*^2^ > 2σ(*F*^2^)] = 0.057	Hydrogen site location: inferred from neighbouring sites
*wR*(*F*^2^) = 0.140	H-atom parameters constrained
*S* = 0.97	*w* = 1/[σ^2^(*F*_o_^2^) + (0.0665*P*)^2^] where *P* = (*F*_o_^2^ + 2*F*_c_^2^)/3
10985 reflections	(Δ/σ)_max_ = 0.001
595 parameters	Δρ_max_ = 0.83 e Å^−^^3^
0 restraints	Δρ_min_ = −0.82 e Å^−^^3^

### Special details

**Table:**

Geometry. All e.s.d.'s (except the e.s.d. in the dihedral angle between two l.s. planes) are estimated using the full covariance matrix. The cell e.s.d.'s are taken into account individually in the estimation of e.s.d.'s in distances, angles and torsion angles; correlations between e.s.d.'s in cell parameters are only used when they are defined by crystal symmetry. An approximate (isotropic) treatment of cell e.s.d.'s is used for estimating e.s.d.'s involving l.s. planes.
Refinement. Refinement of *F*^2^ against ALL reflections. The weighted *R*-factor *wR* and goodness of fit *S* are based on *F*^2^, conventional *R*-factors *R* are based on *F*, with *F* set to zero for negative *F*^2^. The threshold expression of *F*^2^ > σ(*F*^2^) is used only for calculating *R*-factors(gt) *etc*. and is not relevant to the choice of reflections for refinement. *R*-factors based on *F*^2^ are statistically about twice as large as those based on *F*, and *R*- factors based on ALL data will be even larger.

### Fractional atomic coordinates and isotropic or equivalent isotropic displacement parameters (Å^2^)

**Table:**

	*x*	*y*	*z*	*U*_iso_*/*U*_eq_	
Ru1	0.14140 (2)	0.117675 (14)	0.61008 (2)	0.03605 (11)	
Co1	0.39049 (4)	0.07678 (2)	0.78319 (4)	0.03828 (15)	
P1	0.19282 (7)	0.16321 (4)	0.74190 (7)	0.0360 (2)	
P2	0.20918 (7)	0.02449 (4)	0.63204 (7)	0.0337 (2)	
C1	0.3097 (3)	0.15053 (16)	0.7960 (3)	0.0380 (9)	
C2	0.3389 (3)	0.12705 (18)	0.8846 (3)	0.0462 (10)	
H2	0.2982	0.1124	0.9319	0.055*	
C3	0.4366 (3)	0.1275 (2)	0.8917 (3)	0.0581 (13)	
H3	0.4751	0.1129	0.9447	0.070*	
C4	0.4696 (3)	0.15178 (19)	0.8100 (3)	0.0567 (12)	
H4	0.5346	0.1569	0.7960	0.068*	
C5	0.3924 (3)	0.16604 (17)	0.7509 (3)	0.0436 (10)	
H5	0.3948	0.1828	0.6884	0.052*	
C6	0.3211 (3)	0.01700 (16)	0.6984 (3)	0.0350 (9)	
C7	0.3373 (3)	−0.00824 (17)	0.7877 (3)	0.0422 (10)	
H7	0.2898	−0.0221	0.8287	0.051*	
C8	0.4345 (3)	−0.00871 (17)	0.8086 (3)	0.0482 (11)	
H8	0.4651	−0.0231	0.8664	0.058*	
C9	0.4790 (3)	0.01508 (19)	0.7328 (3)	0.0477 (11)	
H9	0.5458	0.0202	0.7281	0.057*	
C10	0.4090 (3)	0.03187 (17)	0.6650 (3)	0.0401 (9)	
H10	0.4197	0.0506	0.6050	0.048*	
C11	0.1157 (3)	0.15023 (18)	0.8351 (3)	0.0425 (10)	
C12	0.0433 (3)	0.1898 (2)	0.8526 (3)	0.0553 (12)	
H12	0.0391	0.2268	0.8220	0.066*	
C13	−0.0219 (4)	0.1747 (3)	0.9148 (4)	0.0734 (16)	
H13	−0.0703	0.2010	0.9256	0.088*	
C14	−0.0144 (4)	0.1201 (3)	0.9607 (4)	0.0820 (18)	
H14	−0.0580	0.1098	1.0030	0.098*	
C15	0.0560 (4)	0.0811 (3)	0.9450 (3)	0.0680 (14)	
H15	0.0605	0.0447	0.9774	0.082*	
C16	0.1203 (3)	0.0950 (2)	0.8819 (3)	0.0544 (12)	
H16	0.1671	0.0676	0.8704	0.065*	
C17	0.2041 (3)	0.24636 (16)	0.7336 (3)	0.0400 (9)	
C18	0.2074 (3)	0.27338 (18)	0.6493 (3)	0.0529 (11)	
H18	0.2017	0.2498	0.5960	0.063*	
C19	0.2189 (4)	0.3351 (2)	0.6418 (4)	0.0695 (15)	
H19	0.2202	0.3531	0.5838	0.083*	
C20	0.2283 (4)	0.3701 (2)	0.7201 (4)	0.0629 (13)	
H20	0.2372	0.4117	0.7152	0.076*	
C21	0.2247 (3)	0.34435 (19)	0.8036 (4)	0.0588 (12)	
H21	0.2294	0.3684	0.8563	0.071*	
C22	0.2141 (3)	0.28226 (17)	0.8121 (3)	0.0480 (10)	
H22	0.2137	0.2646	0.8705	0.058*	
C23	0.0623 (3)	−0.01639 (19)	0.7322 (3)	0.0487 (11)	
H23	0.0452	0.0243	0.7325	0.058*	
C24	0.0082 (3)	−0.0594 (2)	0.7768 (3)	0.0597 (13)	
H24	−0.0447	−0.0474	0.8056	0.072*	
C25	0.0341 (4)	−0.1183 (2)	0.7774 (3)	0.0622 (13)	
H25	−0.0009	−0.1469	0.8074	0.075*	
C26	0.1117 (4)	−0.1362 (2)	0.7340 (3)	0.0632 (13)	
H26	0.1286	−0.1769	0.7343	0.076*	
C27	0.1647 (3)	−0.09449 (18)	0.6902 (3)	0.0497 (11)	
H27	0.2174	−0.1072	0.6617	0.060*	
C28	0.1404 (3)	−0.03386 (16)	0.6880 (3)	0.0363 (9)	
C29	0.2455 (3)	−0.01353 (16)	0.5262 (3)	0.0368 (9)	
C30	0.3125 (3)	−0.05909 (19)	0.5276 (3)	0.0496 (11)	
H30	0.3410	−0.0711	0.5836	0.059*	
C31	0.3367 (3)	−0.0864 (2)	0.4472 (3)	0.0578 (12)	
H31	0.3811	−0.1168	0.4489	0.069*	
C32	0.2961 (3)	−0.0690 (2)	0.3658 (3)	0.0576 (13)	
H32	0.3126	−0.0876	0.3114	0.069*	
C33	0.2299 (4)	−0.0238 (2)	0.3626 (3)	0.0573 (13)	
H33	0.2022	−0.0119	0.3061	0.069*	
C34	0.2053 (3)	0.00331 (18)	0.4428 (3)	0.0452 (10)	
H34	0.1606	0.0336	0.4404	0.054*	
C35	0.0200 (3)	0.1807 (2)	0.6062 (3)	0.0556 (12)	
H35	0.0144	0.2174	0.6438	0.067*	
C36	0.0550 (3)	0.1781 (2)	0.5183 (4)	0.0664 (14)	
H36	0.0772	0.2126	0.4829	0.080*	
C37	0.0423 (3)	0.1183 (3)	0.4852 (3)	0.0649 (14)	
H37	0.0552	0.1037	0.4233	0.078*	
C38	0.0024 (3)	0.0849 (2)	0.5544 (4)	0.0599 (13)	
H38	−0.0164	0.0423	0.5493	0.072*	
C39	−0.0132 (3)	0.1223 (2)	0.6284 (4)	0.0565 (12)	
H39	−0.0450	0.1111	0.6841	0.068*	
C40	0.2633 (3)	0.13775 (17)	0.5551 (3)	0.0408 (9)	
C41	0.3350 (3)	0.14961 (19)	0.5204 (3)	0.0479 (10)	
C42	0.4133 (3)	0.16157 (18)	0.4677 (3)	0.0448 (10)	
C43	0.4693 (3)	0.2130 (2)	0.4812 (3)	0.0526 (11)	
H43	0.4600	0.2384	0.5312	0.063*	
C44	0.4361 (3)	0.1232 (2)	0.3974 (3)	0.0560 (12)	
H44	0.4047	0.0866	0.3895	0.067*	
C45	0.5371 (3)	0.2267 (2)	0.4227 (3)	0.0533 (11)	
H45	0.5721	0.2615	0.4335	0.064*	
C46	0.5042 (3)	0.1380 (2)	0.3387 (3)	0.0591 (13)	
H46	0.5160	0.1114	0.2908	0.071*	
C47	0.5560 (3)	0.19053 (17)	0.3475 (3)	0.0458 (10)	
C48	0.6209 (3)	0.20920 (19)	0.2776 (3)	0.0468 (10)	
C49	0.6845 (3)	0.2560 (2)	0.2922 (3)	0.0519 (11)	
H49	0.6908	0.2740	0.3502	0.062*	
C50	0.6177 (4)	0.1824 (2)	0.1926 (3)	0.0635 (13)	
H50	0.5778	0.1499	0.1812	0.076*	
C51	0.7380 (3)	0.2762 (2)	0.2233 (3)	0.0567 (12)	
H51	0.7792	0.3079	0.2352	0.068*	
C52	0.6720 (4)	0.2021 (2)	0.1229 (3)	0.0642 (13)	
H52	0.6680	0.1826	0.0660	0.077*	
C53	0.7318 (3)	0.2501 (2)	0.1363 (3)	0.0556 (12)	
C54	0.7830 (3)	0.2735 (2)	0.0636 (3)	0.0574 (12)	
C55	0.8215 (4)	0.2942 (2)	0.0036 (4)	0.0694 (14)	
H55	0.8526	0.3110	−0.0450	0.083*	
F1	0.2865 (6)	0.6052 (2)	0.5944 (5)	0.277 (5)	
F2	0.3473 (3)	0.5246 (4)	0.6470 (3)	0.229 (4)	
F3	0.2045 (3)	0.5287 (2)	0.6172 (3)	0.1314 (16)	
F4	0.2322 (3)	0.5580 (3)	0.4767 (3)	0.187 (3)	
F5	0.2957 (5)	0.4787 (2)	0.5262 (4)	0.210 (3)	
F6	0.3773 (3)	0.5565 (3)	0.5076 (3)	0.167 (2)	
P3	0.29026 (9)	0.54231 (6)	0.56284 (9)	0.0591 (3)	

### Atomic displacement parameters (Å^2^)

**Table:**

	*U*^11^	*U*^22^	*U*^33^	*U*^12^	*U*^13^	*U*^23^
Ru1	0.0413 (2)	0.03140 (18)	0.03518 (18)	0.00285 (14)	−0.00097 (13)	−0.00073 (13)
Co1	0.0426 (3)	0.0310 (3)	0.0405 (3)	−0.0005 (2)	−0.0048 (2)	−0.0036 (2)
P1	0.0453 (6)	0.0263 (5)	0.0366 (6)	0.0007 (4)	0.0034 (4)	−0.0005 (4)
P2	0.0384 (6)	0.0290 (5)	0.0334 (5)	−0.0014 (4)	−0.0001 (4)	−0.0019 (4)
C1	0.048 (2)	0.0259 (19)	0.039 (2)	0.0005 (17)	−0.0038 (18)	−0.0064 (16)
C2	0.056 (3)	0.045 (2)	0.036 (2)	0.000 (2)	−0.004 (2)	−0.0086 (18)
C3	0.061 (3)	0.053 (3)	0.058 (3)	0.005 (2)	−0.020 (2)	−0.019 (2)
C4	0.057 (3)	0.040 (3)	0.072 (3)	−0.008 (2)	−0.007 (3)	−0.013 (2)
C5	0.050 (3)	0.028 (2)	0.052 (3)	−0.0030 (18)	0.000 (2)	−0.0036 (18)
C6	0.043 (2)	0.0270 (19)	0.035 (2)	0.0031 (17)	0.0010 (17)	−0.0065 (16)
C7	0.053 (3)	0.031 (2)	0.042 (2)	−0.0039 (19)	−0.0028 (19)	−0.0014 (17)
C8	0.055 (3)	0.033 (2)	0.055 (3)	0.008 (2)	−0.013 (2)	−0.0024 (19)
C9	0.039 (2)	0.042 (2)	0.061 (3)	0.0063 (19)	−0.001 (2)	−0.005 (2)
C10	0.048 (2)	0.040 (2)	0.033 (2)	−0.0005 (19)	0.0064 (18)	−0.0051 (17)
C11	0.051 (3)	0.038 (2)	0.039 (2)	−0.007 (2)	0.0022 (19)	−0.0083 (18)
C12	0.058 (3)	0.052 (3)	0.057 (3)	−0.001 (2)	0.011 (2)	−0.011 (2)
C13	0.058 (3)	0.085 (4)	0.079 (4)	−0.002 (3)	0.020 (3)	−0.028 (3)
C14	0.083 (4)	0.107 (5)	0.058 (3)	−0.036 (4)	0.022 (3)	−0.006 (3)
C15	0.080 (4)	0.068 (3)	0.057 (3)	−0.020 (3)	0.013 (3)	0.006 (3)
C16	0.065 (3)	0.049 (3)	0.051 (3)	−0.006 (2)	0.014 (2)	0.000 (2)
C17	0.049 (2)	0.0261 (19)	0.045 (2)	−0.0007 (17)	0.0024 (19)	−0.0019 (17)
C18	0.076 (3)	0.035 (2)	0.048 (3)	0.000 (2)	0.009 (2)	0.002 (2)
C19	0.104 (4)	0.043 (3)	0.062 (3)	0.004 (3)	0.011 (3)	0.015 (2)
C20	0.082 (4)	0.029 (2)	0.078 (4)	−0.004 (2)	0.007 (3)	−0.002 (2)
C21	0.070 (3)	0.036 (2)	0.070 (3)	0.001 (2)	−0.005 (3)	−0.007 (2)
C22	0.061 (3)	0.030 (2)	0.052 (3)	0.000 (2)	−0.003 (2)	−0.0024 (19)
C23	0.059 (3)	0.038 (2)	0.050 (3)	−0.006 (2)	0.008 (2)	0.000 (2)
C24	0.065 (3)	0.059 (3)	0.057 (3)	−0.006 (3)	0.019 (2)	0.006 (2)
C25	0.076 (4)	0.052 (3)	0.059 (3)	−0.025 (3)	0.002 (3)	0.015 (2)
C26	0.075 (4)	0.041 (3)	0.074 (3)	0.001 (2)	0.003 (3)	0.010 (2)
C27	0.054 (3)	0.038 (2)	0.057 (3)	0.001 (2)	0.006 (2)	0.004 (2)
C28	0.042 (2)	0.033 (2)	0.033 (2)	−0.0034 (17)	−0.0030 (17)	0.0000 (16)
C29	0.045 (2)	0.0270 (19)	0.039 (2)	−0.0061 (17)	0.0075 (18)	−0.0035 (16)
C30	0.057 (3)	0.046 (2)	0.045 (3)	0.012 (2)	0.000 (2)	−0.003 (2)
C31	0.063 (3)	0.052 (3)	0.060 (3)	0.004 (2)	0.011 (2)	−0.013 (2)
C32	0.079 (4)	0.047 (3)	0.049 (3)	−0.015 (3)	0.024 (3)	−0.015 (2)
C33	0.087 (4)	0.050 (3)	0.035 (2)	−0.015 (3)	0.008 (2)	−0.002 (2)
C34	0.062 (3)	0.035 (2)	0.040 (2)	−0.002 (2)	0.003 (2)	−0.0006 (18)
C35	0.053 (3)	0.048 (3)	0.065 (3)	0.016 (2)	−0.007 (2)	−0.004 (2)
C36	0.056 (3)	0.070 (4)	0.072 (4)	0.018 (3)	−0.005 (3)	0.032 (3)
C37	0.063 (3)	0.092 (4)	0.038 (3)	0.025 (3)	−0.013 (2)	−0.006 (3)
C38	0.047 (3)	0.053 (3)	0.077 (4)	0.002 (2)	−0.023 (2)	−0.012 (3)
C39	0.041 (3)	0.060 (3)	0.068 (3)	0.007 (2)	−0.004 (2)	−0.004 (3)
C40	0.054 (3)	0.030 (2)	0.037 (2)	0.0024 (19)	0.000 (2)	−0.0008 (17)
C41	0.053 (3)	0.042 (2)	0.049 (3)	0.001 (2)	0.002 (2)	0.003 (2)
C42	0.046 (2)	0.038 (2)	0.050 (3)	0.0045 (19)	0.001 (2)	0.0121 (19)
C43	0.060 (3)	0.048 (3)	0.050 (3)	−0.007 (2)	0.008 (2)	0.000 (2)
C44	0.059 (3)	0.041 (3)	0.070 (3)	−0.002 (2)	0.018 (2)	−0.003 (2)
C45	0.061 (3)	0.043 (3)	0.057 (3)	−0.010 (2)	0.005 (2)	0.003 (2)
C46	0.069 (3)	0.040 (2)	0.071 (3)	0.002 (2)	0.023 (3)	−0.007 (2)
C47	0.051 (3)	0.030 (2)	0.057 (3)	0.0036 (19)	0.008 (2)	0.0073 (19)
C48	0.050 (3)	0.039 (2)	0.051 (3)	0.005 (2)	0.006 (2)	0.002 (2)
C49	0.056 (3)	0.046 (3)	0.054 (3)	0.002 (2)	0.008 (2)	−0.003 (2)
C50	0.087 (4)	0.049 (3)	0.055 (3)	−0.012 (3)	0.008 (3)	0.001 (2)
C51	0.053 (3)	0.042 (3)	0.076 (3)	−0.006 (2)	0.006 (2)	0.002 (2)
C52	0.080 (4)	0.060 (3)	0.053 (3)	−0.009 (3)	0.009 (3)	−0.004 (2)
C53	0.056 (3)	0.051 (3)	0.061 (3)	0.006 (2)	0.012 (2)	0.010 (2)
C54	0.052 (3)	0.059 (3)	0.062 (3)	−0.001 (2)	0.014 (2)	0.001 (2)
C55	0.064 (3)	0.073 (4)	0.072 (4)	−0.001 (3)	0.013 (3)	0.003 (3)
F1	0.482 (12)	0.096 (4)	0.273 (8)	−0.076 (6)	0.206 (9)	−0.089 (4)
F2	0.097 (3)	0.505 (12)	0.081 (3)	0.065 (5)	−0.039 (2)	−0.003 (5)
F3	0.096 (3)	0.205 (5)	0.095 (3)	−0.009 (3)	0.025 (2)	0.032 (3)
F4	0.115 (3)	0.350 (8)	0.095 (3)	0.060 (4)	0.009 (3)	0.076 (4)
F5	0.354 (9)	0.087 (3)	0.196 (5)	0.015 (4)	0.078 (6)	−0.036 (3)
F6	0.098 (3)	0.277 (6)	0.130 (4)	−0.060 (4)	0.047 (3)	−0.045 (4)
P3	0.0619 (8)	0.0657 (9)	0.0492 (7)	−0.0020 (7)	−0.0004 (6)	−0.0108 (6)

### Geometric parameters (Å, º)

**Table:**

Ru1—C40	2.022 (4)	C23—C28	1.383 (6)
Ru1—C36	2.222 (4)	C23—C24	1.408 (6)
Ru1—C35	2.240 (4)	C23—H23	0.9300
Ru1—C38	2.248 (4)	C24—C25	1.353 (6)
Ru1—C37	2.249 (4)	C24—H24	0.9300
Ru1—P1	2.2497 (12)	C25—C26	1.374 (7)
Ru1—C39	2.271 (5)	C25—H25	0.9300
Ru1—P2	2.2924 (12)	C26—C27	1.373 (6)
Co1—C10	2.008 (4)	C26—H26	0.9300
Co1—C2	2.014 (4)	C27—C28	1.383 (5)
Co1—C3	2.015 (4)	C27—H27	0.9300
Co1—C8	2.017 (4)	C29—C34	1.365 (5)
Co1—C1	2.018 (4)	C29—C30	1.396 (5)
Co1—C5	2.024 (4)	C30—C31	1.372 (6)
Co1—C7	2.029 (4)	C30—H30	0.9300
Co1—C9	2.030 (4)	C31—C32	1.346 (6)
Co1—C6	2.032 (4)	C31—H31	0.9300
Co1—C4	2.038 (4)	C32—C33	1.381 (7)
P1—C11	1.818 (4)	C32—H32	0.9300
P1—C17	1.845 (4)	C33—C34	1.370 (6)
P1—C1	1.850 (4)	C33—H33	0.9300
P2—C28	1.840 (4)	C34—H34	0.9300
P2—C29	1.847 (4)	C35—C36	1.395 (7)
P2—C6	1.849 (4)	C35—C39	1.418 (6)
C1—C2	1.427 (5)	C35—H35	0.9800
C1—C5	1.435 (6)	C36—C37	1.412 (7)
C2—C3	1.414 (6)	C36—H36	0.9800
C2—H2	0.9800	C37—C38	1.392 (7)
C3—C4	1.403 (7)	C37—H37	0.9800
C3—H3	0.9800	C38—C39	1.379 (6)
C4—C5	1.408 (6)	C38—H38	0.9800
C4—H4	0.9800	C39—H39	0.9800
C5—H5	0.9800	C40—C41	1.205 (6)
C6—C7	1.416 (5)	C41—C42	1.424 (6)
C6—C10	1.423 (5)	C42—C44	1.378 (6)
C7—C8	1.424 (6)	C42—C43	1.402 (6)
C7—H7	0.9800	C43—C45	1.363 (6)
C8—C9	1.402 (6)	C43—H43	0.9300
C8—H8	0.9800	C44—C46	1.374 (6)
C9—C10	1.423 (5)	C44—H44	0.9300
C9—H9	0.9800	C45—C47	1.388 (6)
C10—H10	0.9800	C45—H45	0.9300
C11—C16	1.393 (6)	C46—C47	1.383 (6)
C11—C12	1.397 (6)	C46—H46	0.9300
C12—C13	1.379 (7)	C47—C48	1.474 (6)
C12—H12	0.9300	C48—C50	1.365 (6)
C13—C14	1.377 (8)	C48—C49	1.392 (6)
C13—H13	0.9300	C49—C51	1.368 (6)
C14—C15	1.362 (8)	C49—H49	0.9300
C14—H14	0.9300	C50—C52	1.380 (6)
C15—C16	1.374 (6)	C50—H50	0.9300
C15—H15	0.9300	C51—C53	1.384 (6)
C16—H16	0.9300	C51—H51	0.9300
C17—C18	1.362 (5)	C52—C53	1.374 (7)
C17—C22	1.387 (5)	C52—H52	0.9300
C18—C19	1.377 (6)	C53—C54	1.416 (6)
C18—H18	0.9300	C54—C55	1.151 (6)
C19—C20	1.372 (7)	C55—H55	0.9300
C19—H19	0.9300	F1—P3	1.461 (5)
C20—C21	1.339 (6)	F2—P3	1.489 (4)
C20—H20	0.9300	F3—P3	1.534 (4)
C21—C22	1.384 (6)	F4—P3	1.508 (4)
C21—H21	0.9300	F5—P3	1.502 (5)
C22—H22	0.9300	F6—P3	1.558 (4)
			
C40—Ru1—C36	96.27 (18)	C14—C13—C12	119.2 (5)
C40—Ru1—C35	123.65 (17)	C14—C13—H13	120.4
C36—Ru1—C35	36.45 (17)	C12—C13—H13	120.4
C40—Ru1—C38	135.43 (18)	C15—C14—C13	120.8 (5)
C36—Ru1—C38	60.78 (19)	C15—C14—H14	119.6
C35—Ru1—C38	60.20 (17)	C13—C14—H14	119.6
C40—Ru1—C37	102.46 (18)	C14—C15—C16	120.6 (5)
C36—Ru1—C37	36.80 (18)	C14—C15—H15	119.7
C35—Ru1—C37	60.56 (17)	C16—C15—H15	119.7
C38—Ru1—C37	36.06 (17)	C15—C16—C11	120.1 (5)
C40—Ru1—P1	88.78 (11)	C15—C16—H16	119.9
C36—Ru1—P1	113.07 (16)	C11—C16—H16	119.9
C35—Ru1—P1	88.27 (13)	C18—C17—C22	118.6 (4)
C38—Ru1—P1	134.33 (14)	C18—C17—P1	120.0 (3)
C37—Ru1—P1	148.10 (13)	C22—C17—P1	121.3 (3)
C40—Ru1—C39	157.25 (17)	C17—C18—C19	120.9 (4)
C36—Ru1—C39	60.98 (18)	C17—C18—H18	119.6
C35—Ru1—C39	36.65 (16)	C19—C18—H18	119.6
C38—Ru1—C39	35.53 (16)	C20—C19—C18	119.8 (5)
C37—Ru1—C39	60.11 (18)	C20—C19—H19	120.1
P1—Ru1—C39	99.58 (13)	C18—C19—H19	120.1
C40—Ru1—P2	82.80 (11)	C21—C20—C19	120.1 (4)
C36—Ru1—P2	147.61 (16)	C21—C20—H20	119.9
C35—Ru1—P2	152.84 (13)	C19—C20—H20	119.9
C38—Ru1—P2	97.53 (13)	C20—C21—C22	120.6 (4)
C37—Ru1—P2	111.58 (14)	C20—C21—H21	119.7
P1—Ru1—P2	99.30 (4)	C22—C21—H21	119.7
C39—Ru1—P2	116.20 (12)	C21—C22—C17	119.9 (4)
C10—Co1—C2	164.59 (17)	C21—C22—H22	120.0
C10—Co1—C3	152.30 (19)	C17—C22—H22	120.0
C2—Co1—C3	41.09 (18)	C28—C23—C24	120.8 (4)
C10—Co1—C8	68.88 (17)	C28—C23—H23	119.6
C2—Co1—C8	120.47 (18)	C24—C23—H23	119.6
C3—Co1—C8	106.76 (18)	C25—C24—C23	119.4 (5)
C10—Co1—C1	125.43 (16)	C25—C24—H24	120.3
C2—Co1—C1	41.45 (15)	C23—C24—H24	120.3
C3—Co1—C1	69.56 (17)	C24—C25—C26	120.4 (4)
C8—Co1—C1	156.28 (18)	C24—C25—H25	119.8
C10—Co1—C5	106.20 (17)	C26—C25—H25	119.8
C2—Co1—C5	69.07 (17)	C27—C26—C25	120.6 (4)
C3—Co1—C5	68.38 (19)	C27—C26—H26	119.7
C8—Co1—C5	160.57 (17)	C25—C26—H26	119.7
C1—Co1—C5	41.59 (16)	C26—C27—C28	120.8 (4)
C10—Co1—C7	68.86 (16)	C26—C27—H27	119.6
C2—Co1—C7	109.24 (17)	C28—C27—H27	119.6
C3—Co1—C7	126.84 (19)	C27—C28—C23	118.1 (4)
C8—Co1—C7	41.21 (16)	C27—C28—P2	122.9 (3)
C1—Co1—C7	121.26 (17)	C23—C28—P2	118.9 (3)
C5—Co1—C7	156.32 (16)	C34—C29—C30	118.2 (4)
C10—Co1—C9	41.28 (16)	C34—C29—P2	119.0 (3)
C2—Co1—C9	153.59 (17)	C30—C29—P2	122.8 (3)
C3—Co1—C9	117.55 (18)	C31—C30—C29	120.7 (4)
C8—Co1—C9	40.54 (17)	C31—C30—H30	119.6
C1—Co1—C9	162.37 (17)	C29—C30—H30	119.6
C5—Co1—C9	123.54 (18)	C32—C31—C30	120.0 (4)
C7—Co1—C9	68.90 (17)	C32—C31—H31	120.0
C10—Co1—C6	41.26 (15)	C30—C31—H31	120.0
C2—Co1—C6	127.39 (16)	C31—C32—C33	120.4 (4)
C3—Co1—C6	164.92 (19)	C31—C32—H32	119.8
C8—Co1—C6	69.25 (16)	C33—C32—H32	119.8
C1—Co1—C6	107.91 (15)	C34—C33—C32	119.8 (4)
C5—Co1—C6	120.24 (16)	C34—C33—H33	120.1
C7—Co1—C6	40.83 (15)	C32—C33—H33	120.1
C9—Co1—C6	69.50 (16)	C29—C34—C33	120.9 (4)
C10—Co1—C4	117.67 (18)	C29—C34—H34	119.6
C2—Co1—C4	68.91 (19)	C33—C34—H34	119.6
C3—Co1—C4	40.51 (18)	C36—C35—C39	108.2 (4)
C8—Co1—C4	123.68 (19)	C36—C35—Ru1	71.1 (3)
C1—Co1—C4	69.59 (18)	C39—C35—Ru1	72.9 (3)
C5—Co1—C4	40.56 (17)	C36—C35—H35	125.8
C7—Co1—C4	162.61 (18)	C39—C35—H35	125.8
C9—Co1—C4	104.68 (19)	Ru1—C35—H35	125.8
C6—Co1—C4	153.82 (18)	C35—C36—C37	107.5 (5)
C11—P1—C17	105.32 (18)	C35—C36—Ru1	72.5 (3)
C11—P1—C1	104.20 (19)	C37—C36—Ru1	72.7 (3)
C17—P1—C1	95.50 (17)	C35—C36—H36	126.0
C11—P1—Ru1	111.73 (13)	C37—C36—H36	126.0
C17—P1—Ru1	114.52 (13)	Ru1—C36—H36	126.0
C1—P1—Ru1	123.24 (12)	C38—C37—C36	107.5 (4)
C28—P2—C29	103.37 (17)	C38—C37—Ru1	71.9 (3)
C28—P2—C6	100.88 (17)	C36—C37—Ru1	70.5 (3)
C29—P2—C6	96.40 (17)	C38—C37—H37	126.2
C28—P2—Ru1	116.76 (13)	C36—C37—H37	126.2
C29—P2—Ru1	115.46 (13)	Ru1—C37—H37	126.2
C6—P2—Ru1	120.66 (12)	C39—C38—C37	109.6 (4)
C2—C1—C5	106.3 (4)	C39—C38—Ru1	73.1 (3)
C2—C1—P1	131.2 (3)	C37—C38—Ru1	72.0 (3)
C5—C1—P1	122.6 (3)	C39—C38—H38	125.1
C2—C1—Co1	69.1 (2)	C37—C38—H38	125.1
C5—C1—Co1	69.4 (2)	Ru1—C38—H38	125.1
P1—C1—Co1	127.24 (19)	C38—C39—C35	107.1 (5)
C3—C2—C1	108.2 (4)	C38—C39—Ru1	71.4 (3)
C3—C2—Co1	69.5 (2)	C35—C39—Ru1	70.5 (3)
C1—C2—Co1	69.4 (2)	C38—C39—H39	126.4
C3—C2—H2	125.9	C35—C39—H39	126.4
C1—C2—H2	125.9	Ru1—C39—H39	126.4
Co1—C2—H2	125.9	C41—C40—Ru1	178.6 (4)
C4—C3—C2	109.0 (4)	C40—C41—C42	172.2 (5)
C4—C3—Co1	70.6 (3)	C44—C42—C43	116.3 (4)
C2—C3—Co1	69.4 (2)	C44—C42—C41	120.6 (4)
C4—C3—H3	125.5	C43—C42—C41	123.1 (4)
C2—C3—H3	125.5	C45—C43—C42	121.4 (4)
Co1—C3—H3	125.5	C45—C43—H43	119.3
C3—C4—C5	107.6 (4)	C42—C43—H43	119.3
C3—C4—Co1	68.9 (3)	C46—C44—C42	121.1 (4)
C5—C4—Co1	69.2 (2)	C46—C44—H44	119.4
C3—C4—H4	126.2	C42—C44—H44	119.4
C5—C4—H4	126.2	C43—C45—C47	122.6 (4)
Co1—C4—H4	126.2	C43—C45—H45	118.7
C4—C5—C1	109.0 (4)	C47—C45—H45	118.7
C4—C5—Co1	70.3 (2)	C44—C46—C47	123.1 (4)
C1—C5—Co1	69.0 (2)	C44—C46—H46	118.4
C4—C5—H5	125.5	C47—C46—H46	118.4
C1—C5—H5	125.5	C46—C47—C45	115.1 (4)
Co1—C5—H5	125.5	C46—C47—C48	122.2 (4)
C7—C6—C10	107.0 (3)	C45—C47—C48	122.4 (4)
C7—C6—P2	127.5 (3)	C50—C48—C49	116.7 (4)
C10—C6—P2	125.3 (3)	C50—C48—C47	120.3 (4)
C7—C6—Co1	69.5 (2)	C49—C48—C47	122.9 (4)
C10—C6—Co1	68.4 (2)	C51—C49—C48	121.7 (4)
P2—C6—Co1	130.49 (19)	C51—C49—H49	119.1
C6—C7—C8	108.2 (4)	C48—C49—H49	119.1
C6—C7—Co1	69.7 (2)	C48—C50—C52	121.9 (5)
C8—C7—Co1	69.0 (2)	C48—C50—H50	119.0
C6—C7—H7	125.9	C52—C50—H50	119.0
C8—C7—H7	125.9	C49—C51—C53	121.1 (4)
Co1—C7—H7	125.9	C49—C51—H51	119.5
C9—C8—C7	108.7 (4)	C53—C51—H51	119.5
C9—C8—Co1	70.2 (2)	C53—C52—C50	121.2 (5)
C7—C8—Co1	69.8 (2)	C53—C52—H52	119.4
C9—C8—H8	125.7	C50—C52—H52	119.4
C7—C8—H8	125.7	C52—C53—C51	117.3 (4)
Co1—C8—H8	125.7	C52—C53—C54	121.8 (5)
C8—C9—C10	107.3 (4)	C51—C53—C54	120.9 (4)
C8—C9—Co1	69.2 (2)	C55—C54—C53	177.0 (6)
C10—C9—Co1	68.5 (2)	C54—C55—H55	180.0
C8—C9—H9	126.3	F1—P3—F2	91.3 (4)
C10—C9—H9	126.3	F1—P3—F5	177.3 (4)
Co1—C9—H9	126.3	F2—P3—F5	90.4 (4)
C9—C10—C6	108.8 (3)	F1—P3—F4	90.7 (5)
C9—C10—Co1	70.2 (2)	F2—P3—F4	178.0 (4)
C6—C10—Co1	70.3 (2)	F5—P3—F4	87.7 (4)
C9—C10—H10	125.6	F1—P3—F3	88.9 (3)
C6—C10—H10	125.6	F2—P3—F3	87.6 (3)
Co1—C10—H10	125.6	F5—P3—F3	93.2 (3)
C16—C11—C12	118.3 (4)	F4—P3—F3	92.1 (2)
C16—C11—P1	118.9 (3)	F1—P3—F6	90.9 (3)
C12—C11—P1	122.1 (3)	F2—P3—F6	92.5 (3)
C13—C12—C11	120.9 (5)	F5—P3—F6	87.0 (3)
C13—C12—H12	119.6	F4—P3—F6	87.8 (3)
C11—C12—H12	119.6	F3—P3—F6	179.8 (3)
			
C40—Ru1—P1—C11	−170.70 (18)	C6—C7—C8—Co1	58.8 (3)
C36—Ru1—P1—C11	93.0 (2)	C10—Co1—C8—C9	38.0 (2)
C35—Ru1—P1—C11	65.6 (2)	C2—Co1—C8—C9	−155.6 (2)
C38—Ru1—P1—C11	22.0 (2)	C3—Co1—C8—C9	−113.0 (3)
C37—Ru1—P1—C11	77.3 (3)	C1—Co1—C8—C9	169.8 (4)
C39—Ru1—P1—C11	30.58 (19)	C5—Co1—C8—C9	−40.7 (6)
P2—Ru1—P1—C11	−88.19 (15)	C7—Co1—C8—C9	119.6 (4)
C40—Ru1—P1—C17	69.67 (18)	C6—Co1—C8—C9	82.3 (3)
C36—Ru1—P1—C17	−26.6 (2)	C4—Co1—C8—C9	−71.9 (3)
C35—Ru1—P1—C17	−54.06 (19)	C10—Co1—C8—C7	−81.6 (3)
C38—Ru1—P1—C17	−97.6 (2)	C2—Co1—C8—C7	84.7 (3)
C37—Ru1—P1—C17	−42.3 (3)	C3—Co1—C8—C7	127.3 (3)
C39—Ru1—P1—C17	−89.06 (19)	C1—Co1—C8—C7	50.1 (5)
P2—Ru1—P1—C17	152.17 (15)	C5—Co1—C8—C7	−160.3 (5)
C40—Ru1—P1—C1	−45.47 (19)	C9—Co1—C8—C7	−119.6 (4)
C36—Ru1—P1—C1	−141.8 (2)	C6—Co1—C8—C7	−37.3 (2)
C35—Ru1—P1—C1	−169.2 (2)	C4—Co1—C8—C7	168.5 (3)
C38—Ru1—P1—C1	147.2 (2)	C7—C8—C9—C10	1.4 (5)
C37—Ru1—P1—C1	−157.4 (3)	Co1—C8—C9—C10	−58.1 (3)
C39—Ru1—P1—C1	155.8 (2)	C7—C8—C9—Co1	59.5 (3)
P2—Ru1—P1—C1	37.03 (16)	C10—Co1—C9—C8	−119.4 (4)
C40—Ru1—P2—C28	179.47 (17)	C2—Co1—C9—C8	53.1 (5)
C36—Ru1—P2—C28	−90.2 (3)	C3—Co1—C9—C8	83.7 (3)
C35—Ru1—P2—C28	−12.7 (3)	C1—Co1—C9—C8	−166.3 (5)
C38—Ru1—P2—C28	−45.49 (19)	C5—Co1—C9—C8	164.9 (2)
C37—Ru1—P2—C28	−79.9 (2)	C7—Co1—C9—C8	−37.9 (2)
P1—Ru1—P2—C28	91.89 (14)	C6—Co1—C9—C8	−81.7 (3)
C39—Ru1—P2—C28	−13.7 (2)	C4—Co1—C9—C8	125.1 (3)
C40—Ru1—P2—C29	−58.74 (18)	C2—Co1—C9—C10	172.6 (3)
C36—Ru1—P2—C29	31.6 (3)	C3—Co1—C9—C10	−156.9 (3)
C35—Ru1—P2—C29	109.1 (3)	C8—Co1—C9—C10	119.4 (4)
C38—Ru1—P2—C29	76.3 (2)	C1—Co1—C9—C10	−46.9 (6)
C37—Ru1—P2—C29	41.9 (2)	C5—Co1—C9—C10	−75.6 (3)
P1—Ru1—P2—C29	−146.32 (14)	C7—Co1—C9—C10	81.6 (3)
C39—Ru1—P2—C29	108.1 (2)	C6—Co1—C9—C10	37.8 (2)
C40—Ru1—P2—C6	56.46 (18)	C4—Co1—C9—C10	−115.4 (3)
C36—Ru1—P2—C6	146.8 (3)	C8—C9—C10—C6	−1.3 (4)
C35—Ru1—P2—C6	−135.7 (3)	Co1—C9—C10—C6	−59.9 (3)
C38—Ru1—P2—C6	−168.5 (2)	C8—C9—C10—Co1	58.6 (3)
C37—Ru1—P2—C6	157.1 (2)	C7—C6—C10—C9	0.8 (4)
P1—Ru1—P2—C6	−31.11 (15)	P2—C6—C10—C9	−175.0 (3)
C39—Ru1—P2—C6	−136.7 (2)	Co1—C6—C10—C9	59.8 (3)
C11—P1—C1—C2	9.0 (4)	C7—C6—C10—Co1	−59.0 (2)
C17—P1—C1—C2	116.4 (4)	P2—C6—C10—Co1	125.2 (3)
Ru1—P1—C1—C2	−119.5 (3)	C2—Co1—C10—C9	−167.5 (6)
C11—P1—C1—C5	−169.1 (3)	C3—Co1—C10—C9	48.6 (5)
C17—P1—C1—C5	−61.8 (3)	C8—Co1—C10—C9	−37.4 (2)
Ru1—P1—C1—C5	62.4 (3)	C1—Co1—C10—C9	164.3 (2)
C11—P1—C1—Co1	103.1 (3)	C5—Co1—C10—C9	122.8 (3)
C17—P1—C1—Co1	−149.6 (3)	C7—Co1—C10—C9	−81.7 (3)
Ru1—P1—C1—Co1	−25.4 (3)	C6—Co1—C10—C9	−119.6 (3)
C10—Co1—C1—C2	169.0 (2)	C4—Co1—C10—C9	80.6 (3)
C3—Co1—C1—C2	−37.5 (3)	C2—Co1—C10—C6	−47.9 (7)
C8—Co1—C1—C2	47.7 (5)	C3—Co1—C10—C6	168.1 (4)
C5—Co1—C1—C2	−117.6 (3)	C8—Co1—C10—C6	82.2 (2)
C7—Co1—C1—C2	84.0 (3)	C1—Co1—C10—C6	−76.2 (3)
C9—Co1—C1—C2	−154.7 (5)	C5—Co1—C10—C6	−117.7 (2)
C6—Co1—C1—C2	126.7 (2)	C7—Co1—C10—C6	37.9 (2)
C4—Co1—C1—C2	−80.9 (3)	C9—Co1—C10—C6	119.6 (3)
C10—Co1—C1—C5	−73.4 (3)	C4—Co1—C10—C6	−159.8 (2)
C2—Co1—C1—C5	117.6 (3)	C17—P1—C11—C16	−155.7 (3)
C3—Co1—C1—C5	80.1 (3)	C1—P1—C11—C16	−55.8 (4)
C8—Co1—C1—C5	165.3 (4)	Ru1—P1—C11—C16	79.4 (3)
C7—Co1—C1—C5	−158.4 (2)	C17—P1—C11—C12	33.6 (4)
C9—Co1—C1—C5	−37.1 (6)	C1—P1—C11—C12	133.5 (3)
C6—Co1—C1—C5	−115.7 (2)	Ru1—P1—C11—C12	−91.3 (3)
C4—Co1—C1—C5	36.7 (2)	C16—C11—C12—C13	0.0 (7)
C10—Co1—C1—P1	42.5 (3)	P1—C11—C12—C13	170.7 (4)
C2—Co1—C1—P1	−126.5 (4)	C11—C12—C13—C14	0.8 (7)
C3—Co1—C1—P1	−164.0 (3)	C12—C13—C14—C15	−0.4 (8)
C8—Co1—C1—P1	−78.8 (5)	C13—C14—C15—C16	−1.0 (8)
C5—Co1—C1—P1	115.9 (4)	C14—C15—C16—C11	1.8 (8)
C7—Co1—C1—P1	−42.6 (3)	C12—C11—C16—C15	−1.3 (7)
C9—Co1—C1—P1	78.8 (6)	P1—C11—C16—C15	−172.4 (4)
C6—Co1—C1—P1	0.2 (3)	C11—P1—C17—C18	−141.5 (4)
C4—Co1—C1—P1	152.6 (3)	C1—P1—C17—C18	112.2 (4)
C5—C1—C2—C3	−1.0 (4)	Ru1—P1—C17—C18	−18.3 (4)
P1—C1—C2—C3	−179.3 (3)	C11—P1—C17—C22	41.9 (4)
Co1—C1—C2—C3	58.8 (3)	C1—P1—C17—C22	−64.5 (4)
C5—C1—C2—Co1	−59.8 (2)	Ru1—P1—C17—C22	165.0 (3)
P1—C1—C2—Co1	121.8 (3)	C22—C17—C18—C19	−1.0 (7)
C10—Co1—C2—C3	−155.4 (6)	P1—C17—C18—C19	−177.8 (4)
C8—Co1—C2—C3	80.5 (3)	C17—C18—C19—C20	0.8 (8)
C1—Co1—C2—C3	−119.7 (4)	C18—C19—C20—C21	−1.2 (8)
C5—Co1—C2—C3	−80.7 (3)	C19—C20—C21—C22	1.8 (8)
C7—Co1—C2—C3	124.5 (3)	C20—C21—C22—C17	−2.1 (7)
C9—Co1—C2—C3	43.4 (5)	C18—C17—C22—C21	1.7 (7)
C6—Co1—C2—C3	166.6 (3)	P1—C17—C22—C21	178.4 (4)
C4—Co1—C2—C3	−37.1 (3)	C28—C23—C24—C25	1.0 (7)
C10—Co1—C2—C1	−35.7 (7)	C23—C24—C25—C26	−0.7 (8)
C3—Co1—C2—C1	119.7 (4)	C24—C25—C26—C27	0.6 (8)
C8—Co1—C2—C1	−159.8 (2)	C25—C26—C27—C28	−0.7 (7)
C5—Co1—C2—C1	39.0 (2)	C26—C27—C28—C23	0.9 (6)
C7—Co1—C2—C1	−115.8 (2)	C26—C27—C28—P2	179.0 (3)
C9—Co1—C2—C1	163.1 (4)	C24—C23—C28—C27	−1.1 (6)
C6—Co1—C2—C1	−73.7 (3)	C24—C23—C28—P2	−179.2 (3)
C4—Co1—C2—C1	82.6 (3)	C29—P2—C28—C27	39.7 (4)
C1—C2—C3—C4	1.0 (5)	C6—P2—C28—C27	−59.7 (4)
Co1—C2—C3—C4	59.8 (3)	Ru1—P2—C28—C27	167.6 (3)
C1—C2—C3—Co1	−58.8 (3)	C29—P2—C28—C23	−142.2 (3)
C10—Co1—C3—C4	46.3 (5)	C6—P2—C28—C23	118.4 (3)
C2—Co1—C3—C4	−120.0 (4)	Ru1—P2—C28—C23	−14.3 (4)
C8—Co1—C3—C4	122.6 (3)	C28—P2—C29—C34	107.2 (3)
C1—Co1—C3—C4	−82.1 (3)	C6—P2—C29—C34	−150.0 (3)
C5—Co1—C3—C4	−37.4 (3)	Ru1—P2—C29—C34	−21.5 (4)
C7—Co1—C3—C4	163.5 (3)	C28—P2—C29—C30	−72.7 (4)
C9—Co1—C3—C4	80.2 (3)	C6—P2—C29—C30	30.1 (4)
C6—Co1—C3—C4	−165.1 (6)	Ru1—P2—C29—C30	158.6 (3)
C10—Co1—C3—C2	166.2 (3)	C34—C29—C30—C31	−0.4 (6)
C8—Co1—C3—C2	−117.4 (3)	P2—C29—C30—C31	179.5 (3)
C1—Co1—C3—C2	37.8 (3)	C29—C30—C31—C32	0.4 (7)
C5—Co1—C3—C2	82.5 (3)	C30—C31—C32—C33	0.0 (7)
C7—Co1—C3—C2	−76.5 (3)	C31—C32—C33—C34	−0.3 (7)
C9—Co1—C3—C2	−159.9 (3)	C30—C29—C34—C33	0.1 (6)
C6—Co1—C3—C2	−45.2 (8)	P2—C29—C34—C33	−179.8 (3)
C4—Co1—C3—C2	120.0 (4)	C32—C33—C34—C29	0.2 (7)
C2—C3—C4—C5	−0.6 (5)	C40—Ru1—C35—C36	47.1 (4)
Co1—C3—C4—C5	58.5 (3)	C38—Ru1—C35—C36	−80.1 (3)
C2—C3—C4—Co1	−59.1 (3)	C37—Ru1—C35—C36	−38.4 (3)
C10—Co1—C4—C3	−157.7 (3)	P1—Ru1—C35—C36	134.5 (3)
C2—Co1—C4—C3	37.6 (3)	C39—Ru1—C35—C36	−116.9 (4)
C8—Co1—C4—C3	−75.7 (3)	P2—Ru1—C35—C36	−118.3 (4)
C1—Co1—C4—C3	82.1 (3)	C40—Ru1—C35—C39	164.0 (3)
C5—Co1—C4—C3	119.7 (4)	C36—Ru1—C35—C39	116.9 (4)
C7—Co1—C4—C3	−49.5 (7)	C38—Ru1—C35—C39	36.7 (3)
C9—Co1—C4—C3	−115.4 (3)	C37—Ru1—C35—C39	78.5 (3)
C6—Co1—C4—C3	171.3 (3)	P1—Ru1—C35—C39	−108.6 (3)
C10—Co1—C4—C5	82.6 (3)	P2—Ru1—C35—C39	−1.5 (5)
C2—Co1—C4—C5	−82.1 (3)	C39—C35—C36—C37	0.9 (5)
C3—Co1—C4—C5	−119.7 (4)	Ru1—C35—C36—C37	64.7 (3)
C8—Co1—C4—C5	164.6 (3)	C39—C35—C36—Ru1	−63.8 (3)
C1—Co1—C4—C5	−37.6 (3)	C40—Ru1—C36—C35	−142.2 (3)
C7—Co1—C4—C5	−169.2 (5)	C38—Ru1—C36—C35	78.4 (3)
C9—Co1—C4—C5	124.9 (3)	C37—Ru1—C36—C35	115.4 (4)
C6—Co1—C4—C5	51.6 (5)	P1—Ru1—C36—C35	−50.8 (3)
C3—C4—C5—C1	0.0 (5)	C39—Ru1—C36—C35	37.5 (3)
Co1—C4—C5—C1	58.2 (3)	P2—Ru1—C36—C35	131.4 (3)
C3—C4—C5—Co1	−58.3 (3)	C40—Ru1—C36—C37	102.4 (3)
C2—C1—C5—C4	0.6 (4)	C35—Ru1—C36—C37	−115.4 (4)
P1—C1—C5—C4	179.2 (3)	C38—Ru1—C36—C37	−37.0 (3)
Co1—C1—C5—C4	−59.0 (3)	P1—Ru1—C36—C37	−166.2 (3)
C2—C1—C5—Co1	59.6 (3)	C39—Ru1—C36—C37	−77.9 (3)
P1—C1—C5—Co1	−121.8 (3)	P2—Ru1—C36—C37	16.0 (5)
C10—Co1—C5—C4	−113.9 (3)	C35—C36—C37—C38	−1.8 (5)
C2—Co1—C5—C4	81.6 (3)	Ru1—C36—C37—C38	62.8 (3)
C3—Co1—C5—C4	37.4 (3)	C35—C36—C37—Ru1	−64.6 (3)
C8—Co1—C5—C4	−41.6 (6)	C40—Ru1—C37—C38	159.4 (3)
C1—Co1—C5—C4	120.5 (4)	C36—Ru1—C37—C38	−116.8 (4)
C7—Co1—C5—C4	172.0 (4)	C35—Ru1—C37—C38	−78.8 (3)
C9—Co1—C5—C4	−72.1 (3)	P1—Ru1—C37—C38	−92.3 (4)
C6—Co1—C5—C4	−156.4 (3)	C39—Ru1—C37—C38	−36.4 (3)
C10—Co1—C5—C1	125.6 (2)	P2—Ru1—C37—C38	72.3 (3)
C2—Co1—C5—C1	−38.9 (2)	C40—Ru1—C37—C36	−83.8 (3)
C3—Co1—C5—C1	−83.2 (3)	C35—Ru1—C37—C36	38.0 (3)
C8—Co1—C5—C1	−162.1 (5)	C38—Ru1—C37—C36	116.8 (4)
C7—Co1—C5—C1	51.4 (5)	P1—Ru1—C37—C36	24.5 (5)
C9—Co1—C5—C1	167.3 (2)	C39—Ru1—C37—C36	80.5 (3)
C6—Co1—C5—C1	83.1 (3)	P2—Ru1—C37—C36	−170.9 (3)
C4—Co1—C5—C1	−120.5 (4)	C36—C37—C38—C39	2.0 (5)
C28—P2—C6—C7	−21.7 (4)	Ru1—C37—C38—C39	63.9 (3)
C29—P2—C6—C7	−126.7 (3)	C36—C37—C38—Ru1	−61.9 (3)
Ru1—P2—C6—C7	108.6 (3)	C40—Ru1—C38—C39	−147.1 (3)
C28—P2—C6—C10	153.3 (3)	C36—Ru1—C38—C39	−80.0 (3)
C29—P2—C6—C10	48.3 (3)	C35—Ru1—C38—C39	−37.9 (3)
Ru1—P2—C6—C10	−76.4 (3)	C37—Ru1—C38—C39	−117.8 (4)
C28—P2—C6—Co1	−116.0 (3)	P1—Ru1—C38—C39	14.6 (4)
C29—P2—C6—Co1	139.0 (3)	P2—Ru1—C38—C39	125.5 (3)
Ru1—P2—C6—Co1	14.3 (3)	C40—Ru1—C38—C37	−29.3 (4)
C10—Co1—C6—C7	118.9 (3)	C36—Ru1—C38—C37	37.8 (3)
C2—Co1—C6—C7	−75.5 (3)	C35—Ru1—C38—C37	79.9 (3)
C3—Co1—C6—C7	−39.6 (7)	P1—Ru1—C38—C37	132.4 (3)
C8—Co1—C6—C7	37.6 (2)	C39—Ru1—C38—C37	117.8 (4)
C1—Co1—C6—C7	−117.4 (2)	P2—Ru1—C38—C37	−116.7 (3)
C5—Co1—C6—C7	−161.2 (2)	C37—C38—C39—C35	−1.5 (5)
C9—Co1—C6—C7	81.1 (3)	Ru1—C38—C39—C35	61.8 (3)
C4—Co1—C6—C7	162.6 (4)	C37—C38—C39—Ru1	−63.2 (3)
C2—Co1—C6—C10	165.6 (2)	C36—C35—C39—C38	0.3 (5)
C3—Co1—C6—C10	−158.5 (6)	Ru1—C35—C39—C38	−62.3 (3)
C8—Co1—C6—C10	−81.2 (3)	C36—C35—C39—Ru1	62.7 (3)
C1—Co1—C6—C10	123.7 (2)	C40—Ru1—C39—C38	80.3 (5)
C5—Co1—C6—C10	79.9 (3)	C36—Ru1—C39—C38	79.4 (3)
C7—Co1—C6—C10	−118.9 (3)	C35—Ru1—C39—C38	116.7 (4)
C9—Co1—C6—C10	−37.8 (2)	C37—Ru1—C39—C38	36.9 (3)
C4—Co1—C6—C10	43.7 (5)	P1—Ru1—C39—C38	−169.4 (3)
C10—Co1—C6—P2	−118.7 (4)	P2—Ru1—C39—C38	−64.0 (3)
C2—Co1—C6—P2	46.9 (3)	C40—Ru1—C39—C35	−36.4 (6)
C3—Co1—C6—P2	82.8 (7)	C36—Ru1—C39—C35	−37.3 (3)
C8—Co1—C6—P2	160.0 (3)	C38—Ru1—C39—C35	−116.7 (4)
C1—Co1—C6—P2	5.0 (3)	C37—Ru1—C39—C35	−79.8 (3)
C5—Co1—C6—P2	−38.8 (3)	P1—Ru1—C39—C35	73.8 (3)
C7—Co1—C6—P2	122.4 (4)	P2—Ru1—C39—C35	179.2 (2)
C9—Co1—C6—P2	−156.5 (3)	C44—C42—C43—C45	5.5 (6)
C4—Co1—C6—P2	−75.0 (5)	C41—C42—C43—C45	−172.3 (4)
C10—C6—C7—C8	0.0 (4)	C43—C42—C44—C46	−6.0 (7)
P2—C6—C7—C8	175.7 (3)	C41—C42—C44—C46	171.8 (4)
Co1—C6—C7—C8	−58.4 (3)	C42—C43—C45—C47	−1.0 (7)
C10—C6—C7—Co1	58.4 (2)	C42—C44—C46—C47	2.1 (8)
P2—C6—C7—Co1	−125.9 (3)	C44—C46—C47—C45	2.5 (7)
C10—Co1—C7—C6	−38.3 (2)	C44—C46—C47—C48	−172.5 (5)
C2—Co1—C7—C6	125.4 (2)	C43—C45—C47—C46	−3.1 (7)
C3—Co1—C7—C6	168.0 (2)	C43—C45—C47—C48	172.0 (4)
C8—Co1—C7—C6	−119.9 (4)	C46—C47—C48—C50	14.1 (7)
C1—Co1—C7—C6	81.2 (3)	C45—C47—C48—C50	−160.5 (4)
C5—Co1—C7—C6	43.8 (5)	C46—C47—C48—C49	−169.0 (4)
C9—Co1—C7—C6	−82.7 (2)	C45—C47—C48—C49	16.3 (6)
C4—Co1—C7—C6	−153.8 (6)	C50—C48—C49—C51	3.1 (7)
C10—Co1—C7—C8	81.7 (3)	C47—C48—C49—C51	−173.9 (4)
C2—Co1—C7—C8	−114.6 (3)	C49—C48—C50—C52	−2.6 (7)
C3—Co1—C7—C8	−72.0 (3)	C47—C48—C50—C52	174.5 (4)
C1—Co1—C7—C8	−158.8 (2)	C48—C49—C51—C53	−0.9 (7)
C5—Co1—C7—C8	163.8 (4)	C48—C50—C52—C53	−0.2 (8)
C9—Co1—C7—C8	37.3 (2)	C50—C52—C53—C51	2.5 (7)
C6—Co1—C7—C8	119.9 (4)	C50—C52—C53—C54	−175.5 (5)
C4—Co1—C7—C8	−33.9 (7)	C49—C51—C53—C52	−2.0 (7)
C6—C7—C8—C9	−0.9 (4)	C49—C51—C53—C54	176.1 (4)
Co1—C7—C8—C9	−59.7 (3)		

### Hydrogen-bond geometry (Å, º)

**Table:**

*D*—H···*A*	*D*—H	H···*A*	*D*···*A*	*D*—H···*A*
C2—H2···F5^i^	0.98	2.43	3.189 (6)	134
C3—H3···F6^ii^	0.98	2.54	3.383 (6)	144
C7—H7···F4^i^	0.98	2.47	3.383 (7)	155
C9—H9···F2^ii^	0.98	2.32	2.993 (6)	125

Symmetry codes: (i) *x*, −*y*+1/2, *z*+1/2; (ii) −*x*+1, *y*−1/2, −*z*+3/2.
